# Encephalitis with status epilepticus and stroke as complications of non-severe COVID-19 in a young female patient: a case report

**DOI:** 10.1186/s12883-022-02782-3

**Published:** 2022-07-11

**Authors:** Seungyon Koh, Yoon Seob Kim, Min Hye Kim, Young Hwa Choi, Jun Young Choi, Tae-Joon Kim

**Affiliations:** 1 Department of Neurology, Ajou University School of Medicine, Suwon, Republic of Korea; 2 Department of Brain Science, Ajou University School of Medicine, Suwon, Republic of Korea; 3 Department of Neurology, Ajou University Hospital, Suwon, Republic of Korea; 4 Department of Infectious Disease, Ajou University School of Medicine, Suwon, Republic of Korea

**Keywords:** COVID-19, Status epilepticus, Encephalitis, Stroke, Interleukin-6

## Abstract

**Background:**

Neurological manifestations of COVID-19 are thought to be associated with the disease severity of COVID-19 and poor clinical outcomes. Dysregulated immune responses are considered to be mediating such complications. Our case illustrates multiple critical neurological complications simultaneously developed in a patient with non-severe COVID-19 and successful recovery with a multifaceted therapeutic approach. The cerebrospinal fluid (CSF) interleukin-6 (IL-6) level was temporally correlated with the clinical severity of the status epilepticus in our patient, suggesting a causal relationship.

**Case presentation:**

A previously healthy 20-year-old female patient presented with a first-onset seizure. Concomitant non-severe COVID-19 pneumonia was diagnosed. CSF study showed lymphocytic pleocytosis with elevated IL-6 levels in CSF. During hospitalization under the diagnosis of autoimmune encephalitis, status epilepticus developed, and the seizure frequency was temporally correlated with the CSF IL-6 level. Furthermore, a new embolic stroke developed without a significant cardioembolic source. Contrary to the exacerbated COVID-19-associated neurological complications, COVID-19 pneumonia was cleared entirely. After treatment with antiseizure medications, antithrombotics, antiviral agents, and immunotherapy, the patient was discharged with near-complete recovery.

**Conclusion:**

Active serological, and radiological evaluation can be helpful even in non-severe COVID-19, and multidimensional treatment strategies, including immunotherapy, can successfully reverse the neurological complication.

## Background

Neurological manifestations of coronavirus disease 2019 (COVID-19) can occur, which are known to be associated with disease severity of COVID-19 and poor clinical outcomes [1]. Various neurotropic properties of the SARS-CoV-2, including the hematogenous and neuronal transmission and other neurovirulent mechanisms such as hypoxia-related, inflammation-related, and angiotensin-converting enzyme-2 binding-related neuroinvasion have been previously suggested [2, 3]. Among many neurological syndromes of COVID-19, serious neurological complications such as seizures, encephalitis, and stroke have been previously reported. Seizures accounted for 0.5–1% of the neurological complications and were commonly associated with systemic derangements, suggesting acute symptomatic seizures [4]. Encephalitis showed an incidence rate of 0.25% among all COVID-19 and was associated with significant morbidity and mortality [5]. Ischemic strokes occurring in COVID-19 patients were usually associated with underlying cardiovascular risk factors [6]; however, thrombogenic vasculopathy of COVID-19 has also been suggested in young patients [7].

Herein, we describe a patient with multiple critical neurological complications with concomitant non-severe COVID-19 and successful recovery after a multifaceted treatment strategy.

## Case presentation

Our patient was a previously healthy 20-year-old woman with unremarkable medical/neurological history. She presented with a first-onset focal tonic-clonic seizure on her left face and arm, followed by a drowsy mentality. The family reported that the patient experienced an unusual personality change from a week ago, exhibiting reckless and violent driving. The day before the hospital visit, the patient complained of dizziness and somnolence. She did not have a history of febrile seizures or any family history of seizure disorders. On presentation, she had an unnoticed fever, for which the polymerase chain reaction (PCR) for SARS-CoV-2 on nasal swab was tested and yielded a positive result. Vital signs were normal, with a respiratory rate of 18/min and SpO_2_ of 98% in room air. The arterial blood gas analysis was within the normal range, revealing pH 7.40, pCO2 37.6, pO2 84.6, and HCO3 23.4.

**Fig. 1:**
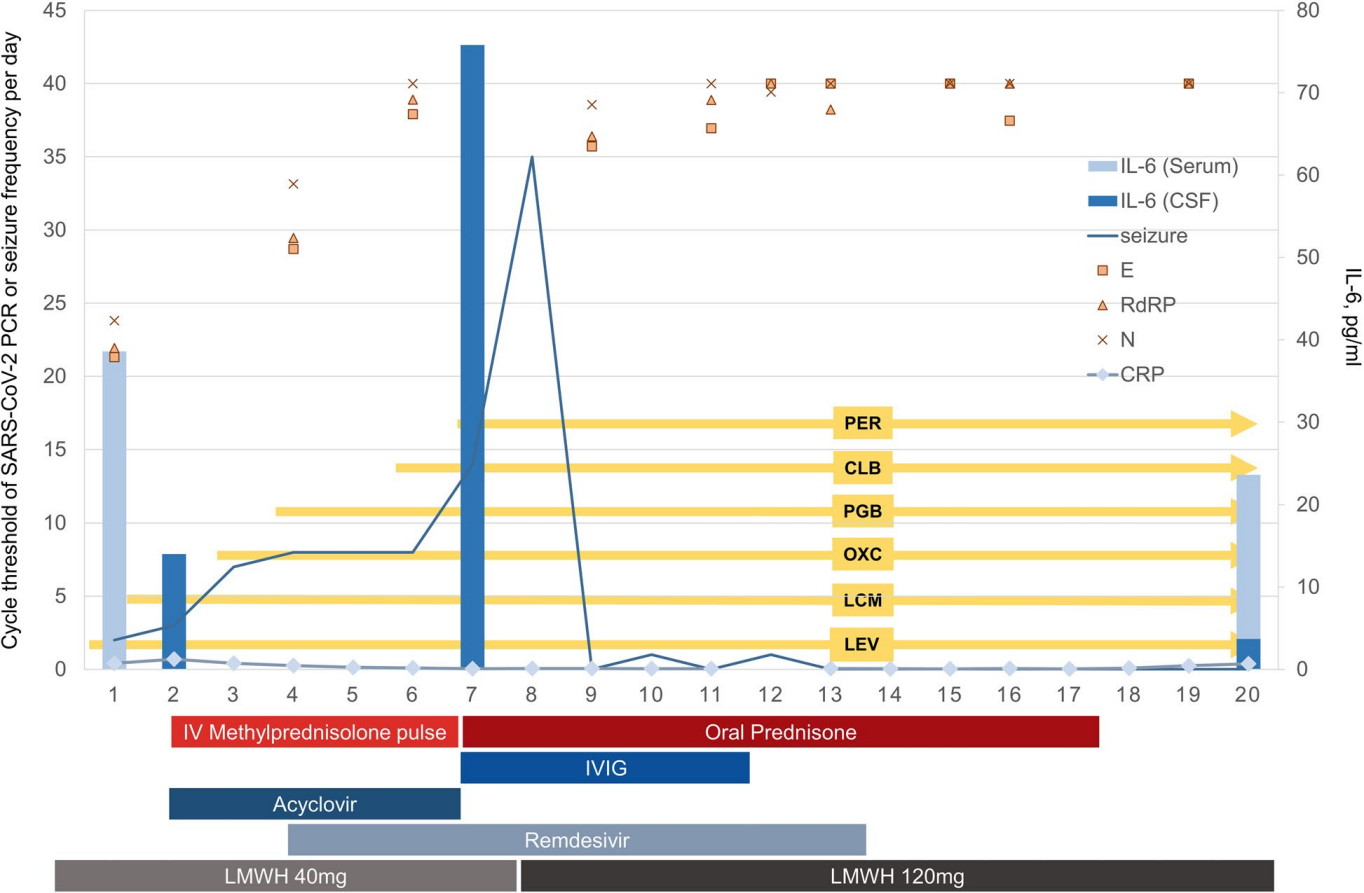
A brief description of the progress of the patient. Note that CSF IL-6 (light blue bar graph) level is temporally correlated with the daily seizure frequency (line graph). SARS-CoV-2 PCR cycle threshold values for each gene (E, RdRP, N) are marked as square, triangle, and cross. Serum CRP level is shown in rhombus with connecting lines and was maintained below 1 mg/dl throughout the treatment period. CLB, clobazam; CRP, C-reactive protein; CSF, cerebrospinal fluid; IL-6, interleukin-6; IV, intravenous; IVIG, intravenous immunoglobulin G; LCM, lacosamide; LEV, levetiracetam; LMWH, low molecular weight heparin; OXC, oxcarbazepine; PCR, polymerase chain reaction; PER, perampanel; PGB, pregabalin

**Fig. 2:**
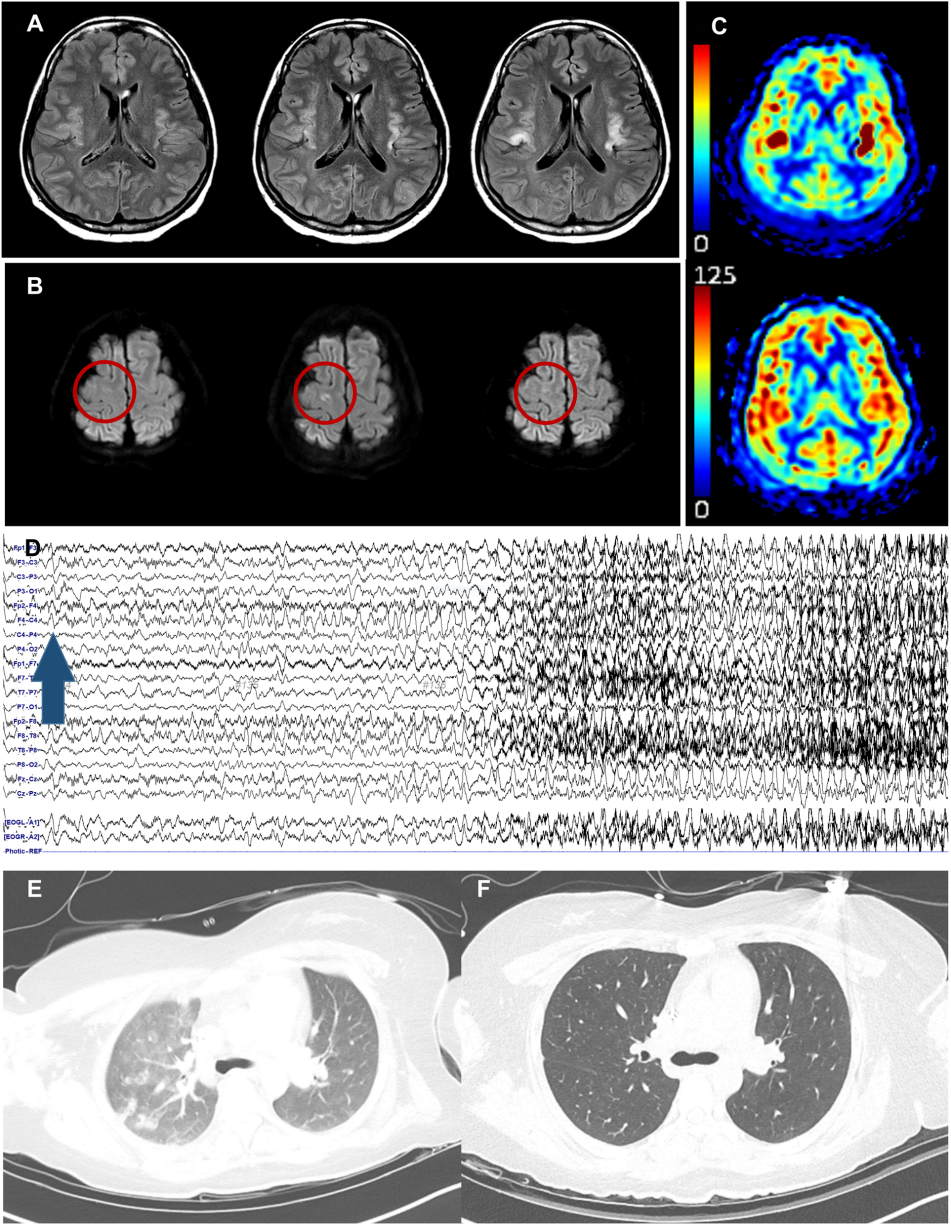
**A**-**C** are magnetic resonance imaging findings along the treatment period. **A** shows fluid-attenuated inversion recovery images at presentation, 7th day, and 15th day, from left to right. Note that high signal intensities involving bilateral insula intensify over time. **B** shows diffusion-weighted imaging at presentation, 7th day, and 15th day, from left to right. The red circle indicates the evolution of ischemic stroke lesion in the right frontal lobe, which developed on the 7th day and normalized on the 15th day. **A** and **B** show different aging stages of each lesion, supporting that the newly developed focal diffusion restriction in **B** is more likely due to an ischemic nature. **C** shows arterial spin labeling signals at presentation and two months after seizure resolution, from top to bottom. Note that the focal increase in bilateral insula, suggestive of ictal perfusion increase, is resolved. **D** shows the electroencephalogram findings of the patient. High amplitude polymorphic delta activities from the right frontotemporal area evolving to generalized 1-2 Hz spike-wave discharges were noted. The blue arrow indicates seizure onset from the right frontal and anterior temporal region. **E** is a chest computed tomography (CT) finding at presentation, which shows patchy ground-glass opacities in the right upper lung field, suggestive of COVID-19 pneumonia. **F** is a follow-up chest CT on day 10. Previously seen COVID-19 pneumonia is completely cleared

The progress of the patient is depicted in Fig. 1. Upon admission, focal seizures persisted, and continuous video-electroencephalogram (EEG) monitoring showed repeated high amplitude polymorphic delta activities from the right frontotemporal area evolving to generalized 1-2 Hz spike-wave discharges, suggesting an impending focal status epilepticus (Fig. 2). A cerebrospinal fluid (CSF) study showed lymphocytic pleocytosis (white blood cells 18 /ul, 93% lymphocyte) with CSF interleukin-6 (IL-6) level of 14.0 pg/ml, while a PCR test for SARS-CoV-2 in CSF was negative. Serum IL-6 was mildly elevated to 21.7 pg/ml, while serum C-reactive protein (CRP) was normal (0.70 mg/dl). Pathogen screening panels for serum and CSF included herpes simplex virus, varicella-zoster virus, enterovirus, tuberculosis, Ebstein-Barr virus, toxoplasmosis, and syphilis which came back negative. Brain magnetic resonance imaging (MRI) showed diffuse cortical high signal intensities, especially on bilateral insula with increased arterial spin labeling signals. Chest computed tomography (CT) showed patchy ground-glass opacities on bilateral lung fields, compatible with COVID-19 pneumonia. Serial images of brain MRI and chest CT are depicted in Fig. 2.

Given the subacute onset of neuropsychiatric symptoms with new-onset focal status epilepticus and CSF pleocytosis, a tentative diagnosis of probable autoimmune encephalitis was made. Antibodies against neuronal synaptic or intraneuronal antigens were negative in serum and CSF, and no solid tumor was found. Extensive screening tests for the autoimmune diseases came back negative as well, including thyroid panel, vasculitis lab, immunoglobulin G subclass, and anti-ganglioside antibodies. Clinical and EEG seizures progressively aggravated up to 35 per day during the first week. Despite the persistent seizures, intravenous (IV) anesthetic therapy was not considered because the seizures remained focal with only occasional generalization, and therefore the vital signs remained intact.

On day 7, a newly developed embolic stroke on the right frontal lobe was detected on a follow-up MRI. Considering the extensive seizure-related changes involving both insula areas, the new isolated focal lesion was thought of as evidence of an embolic stroke. Etiological evaluation of stroke revealed negative results, including transthoracic echocardiogram, electrocardiogram, Holter monitoring, and transcranial Doppler bubble study to rule out patent foramen ovale. Initially elevated D-dimer level (1.48 μg/ml) was decreased to 1.00 μg/ml. Routine thromboprophylaxis using low molecular weight heparin was increased to a therapeutic dose of secondary stroke prevention (2 mg/kg). The follow-up CSF study showed more increased IL-6 level of 75.8 pg/ml. Contrary to the exacerbated COVID-19-associated neurological complications, the follow-up chest CT revealed completely cleared bilateral lung fields. Daily followed serum CRP level was maintained under 1 mg/dl.

Along with IV acyclovir and remdesivir, IV methylprednisolone pulse therapy was given to treat autoimmune encephalitis. Because the seizures persisted after the methylprednisolone, IV immunoglobulin G was administrated next, to which the patient started to respond. The seizures finally halted on the 12th day, and her neurological deficits gradually resolved thereafter. During the course of the treatment, her otherwise vital signs remained stable and intact oxygenation was maintained. A Follow-up study revealed normalized CSF IL-6 level to 3.7 pg/ml and decreased serum IL-6 level to 13.3 pg/ml. After 39 days of hospitalization, the patient successfully recovered from COVID-19 pneumonia, encephalitis, status epilepticus, and stroke, and she was discharged home at a modified Rankin scale of 1.

## Discussion and conclusions

Our case illustrates multiple critical neurological complications simultaneously developed in a patient with non-severe COVID-19 and successful recovery with a multifaceted therapeutic approach. The commonality in the development of the neurological complications observed in our patient can be characterized as dysregulated immune response, which led to thromboinflammation and neuroinflammation [8]. This alteration of immune homeostasis and subsequent pro-inflammatory conditions occurring in COVID-19 were previously thought to be positively correlated with the severity of the infection. However, our case distinctively shows multiple critical neurological manifestations precipitated by non-severe and even resolving state of pulmonary COVID-19.

The CSF IL-6 level was temporally correlated with the clinical severity of the status epilepticus in our patient, and the immunotherapies improved the patient’s condition, suggesting a causal relationship. This association with IL-6 and status epilepticus in our patient also raises the possibility of new-onset refractory status epilepticus (NORSE) diagnosis [9, 10]. However, we postulate that this specific case did not fall under the typical diagnosis of NORSE because of the course of the status epilepticus, which was characterized by rapid cessation without the use of IV anesthetic therapy and focal semiology or electrographic seizures without coma. The serological association between the disease severity of COVID-19 and serum IL-6 level as a key cytokine has been widely studied [11]. What is noteworthy about this particular case is that the dysregulated host response to SARS-CoV-2, represented by elevated CSF IL-6 and clinical manifestations, was confined to the extrapulmonary system, especially to the central nervous system (CNS). Although serum IL-6 level was not measured at the time of the most severe seizures, we speculate that the IL-6-mediated inflammatory response provoked by COVID-19 infection was limited to the CNS, considering the only mildly elevated initial serum IL-6 level while the pulmonary involvement was evident, and constantly normal-ranged other inflammatory markers, such as CRP.

Although we cannot confirm the exact immune mechanism by which the neuroinvasion occurred in this patient, the clear implication of our case is that multiple critical neurological complications by the immune response can occur in non-severe COVID-19. Also, a multidimensional therapeutic approach, especially with immunotherapy despite COVID-19, successfully reversed the neurological complications of COVID-19.

In conclusion, our case exemplifies a variety of severe neurological syndrome occurring in a non-severe COVID-19 patient and signifies an immunological aspect of neurovirulence in COVID-19. Our case thereby suggests that active neurological evaluation and multifaceted treatment can be helpful in COVID-19 patients, despite mild pulmonary manifestation.

## Data Availability

The data supporting this study’s findings are available on request from the corresponding author [TJ Kim]. The data are not publicly available due [to them containing information that could compromise research participant privacy.]

## Abbreviations

CNS: Central nervous system
CRP: C-reactive protein
CSF: Cerebrospinal fluid
CT: Computed tomography
EEG: Electroencephalogram
IL-6: Interleukin-6
IV: Intravenous
MRI: Magnetic resonance imaging
NORSE: New-onset refractory status epilepticus
PCR: Polymerase chain reaction
